# Biliary Adenofibroma with Invasive Carcinoma: Case Report and Review of the Literature

**DOI:** 10.1155/2016/8068513

**Published:** 2016-01-13

**Authors:** Anjali Godambe, Elizabeth M. Brunt, Keith H. Fulling, Taher Reza Kermanshahi

**Affiliations:** ^1^Ameripath Indiana, Indianapolis, IN, USA; ^2^Department of Pathology and Immunology, Washington University, St. Louis, MO, USA; ^3^Mercy Hospital, St. Louis, MO, USA; ^4^Department of Pathology, University of Pittsburgh Medical Center, Pittsburgh, PA, USA

## Abstract

We report a case of biliary adenofibroma with an invasive carcinoma in a 71-year-old female who presented with bilateral upper abdominal pain. Imaging revealed a 6.3 cm heterogeneously enhancing mass in the left lateral segment of the liver. Histologically, the adenofibroma showed the characteristic components as previously described of biliary adenofibromata, namely, cystic and tubular structures lined by cuboidal to low columnar biliary type epithelium and a dense fibrous stroma composed of spindled cells. Intimately admixed with the adenofibroma was a distinct tumor composed of malignant clear cells which demonstrated stromal and vascular invasion. Although mitotic figures were inconspicuous, Ki67 was brisk and p53 demonstrated 25–50% positivity. Sections also showed a von Meyenberg complex located adjacent to the tumor. This case expands the understanding of this rare tumor and proves two important assertions from previous case reports. First, the presence of an associated von Meyenberg complex with similar morphology and immunohistochemical staining pattern suggests that biliary adenofibromata and von Meyenberg complexes may share related histogenesis. Second, biliary adenofibromata harbor malignant potential and may show malignant transformation. Furthermore, this case highlights the need for these rare tumors to be followed aggressively, as their biological behavior is poorly understood.

## 1. Introduction

Biliary adenofibromata are extremely rare tumors with unknown etiology and previously uncertain malignant potential. The tumor was first described by Tsui et al. in 1993 and since then nine cases have been reported in the medical literature. Biliary adenofibromata are characterized by cystic and tubular biliary epithelial components surrounded by a bland fibroblastic spindled stroma [1]. The tumors bear a striking resemblance to bile duct hamartomas (von Meyenberg complexes); a similarity that has been noted in all previously reported cases in the medical literature. Until this report, von Meyenberg complexes have not been identified in histologic sections of tumor or adjacent uninvolved liver.

## 2. Case Report 

A 71-year-old Caucasian female presented with chest pain. Her subsequent workup included a chest CT which revealed a 6.3 cm mass in the right aspect of the left lateral liver straddling segments 2, 3, and 4a. The tumor was best visualized in the precontrast and arterial phases. Contrast enhancement was heterogeneous throughout the mass with multiple areas of low attenuation. Of note, a PET scan showed a 1.9 cm hypermetabolic focus within a 4.3 cm tumor. The patient reported bilateral upper abdominal pain but denied any nausea, vomiting, and weight loss or gain during this period. She had no jaundice, change in bowel habits, or gastrointestinal bleeding. There was no history of significant alcohol or intravenous drug use. The patient had no history of transfusions. She denied personal or family history of liver or biliary disease. Laboratory tests revealed normal liver function testing, serum alpha fetoprotein, CEA, and CA19-9. A biopsy was performed and showed biliary epithelium arranged in cysts and tubules with a fibrotic/spindled stroma. Subsequently, the patient underwent a left hepatectomy. Intraoperative palpation of the abdominal cavity revealed no signs of carcinomatosis. There were no postoperative complications. The patient reported no symptoms after surgery.

### 2.1. Macroscopic Findings

**Figure 1:**
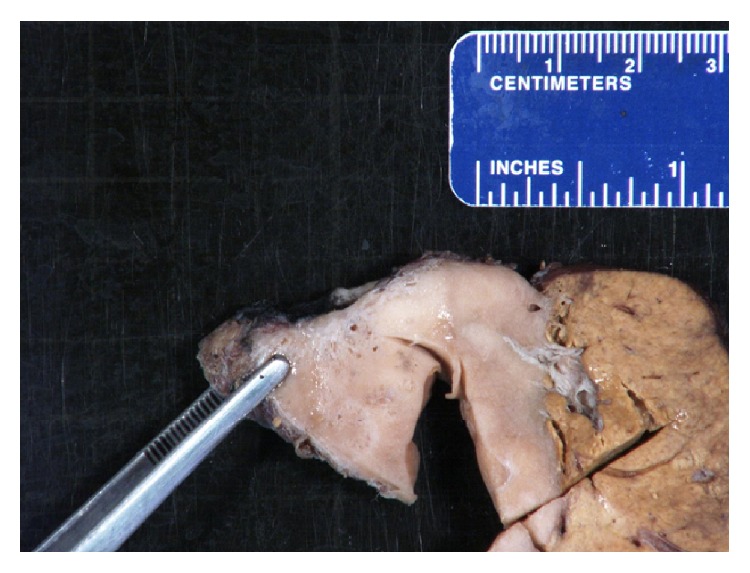
Tumor and surrounding liver parenchyma.

The partial lobectomy from the left lobe contained a circumscribed tumor measuring 5.7 cm that abutted and appeared to invade the liver capsule. The cut surface revealed a smooth tan-white surface with subtle variegation in color and firmness. Small cystic spaces were noted throughout the tumor (Figure 1). The surrounding hepatic parenchyma was unremarkable.

### 2.2. Microscopic Findings

**Figure 2:**
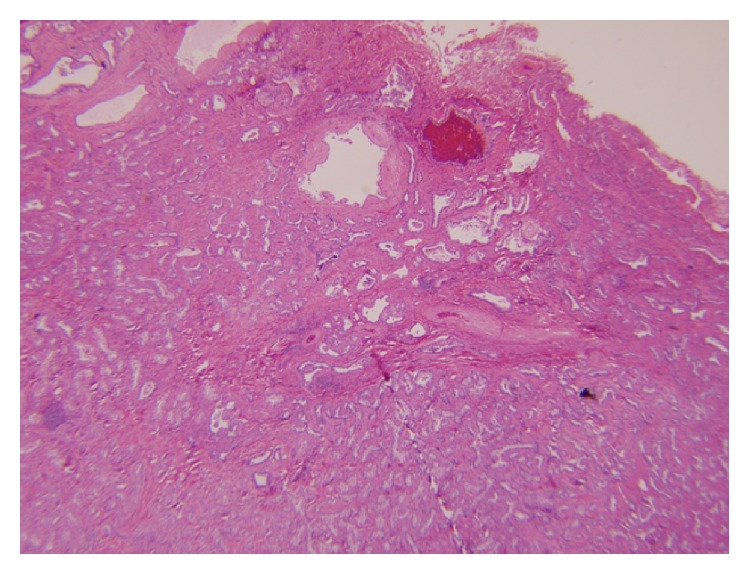
Conventional biliary adenofibroma, H&E 4x.

**Figure 3:**
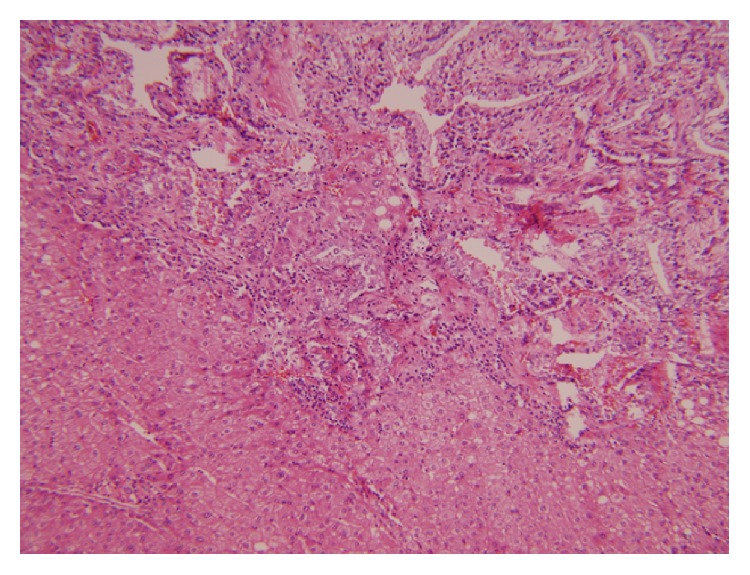
Infiltrative border of tumor to surrounding liver, H&E 10x.

The tumor showed tubulocystic structures embedded in a bland spindled stroma, consistent with a conventional biliary adenofibroma (Figure 2). A distinct carcinomatous component was identified showing solid, glandular, and papillary architecture. The malignant component showed an infiltrative border with invasion into the liver capsule and additionally showed penetration into surrounding adhesions and skeletal muscle. Perineural tumor infiltration, intravenous invasion, and single cell stromal invasion were seen. The tumor was focally seen in the adventitia of a large outflow vein. Although the tumor appeared grossly circumscribed, microscopic examination showed infiltrative margins (Figure 3). The cells of the malignant component showed vacuolated cytoplasm with centrally placed nuclei.

To rule out metastasis from adrenal or renal primary sites, inhibin and PAX8 immunohistochemistries were performed and were negative. A cytokeratin 7 and cytokeratin 19 showed strong positivity in the bland tubulocystic areas of conventional biliary adenofibroma and a lesser degree of positivity in the solid and papillary carcinomatous areas. CD56 stained scattered single cells, similar to the reactivity of the ductular reaction in surrounding nontumor liver. A Ki-67 was uniformly brisk, despite inconspicuous mitotic figures. P53 showed moderate (25–50%) positivity in the tumor. Both Ki67 and p53 were negative in the stromal component. CD10 and polyclonal CEA failed to show either cytoplasmic or canalicular reactivity in tumor cells.

A von Meyenberg complex was seen in hepatic parenchyma adjacent to the tumor.

## 3. Discussion

These cases report the tenth biliary adenofibroma in the medical literature, although two previous reports both from Turkey report identical tumor and patient characteristics and most likely represent the same tumor [2, 3]. The tumor shows a tubulocystic component with a bland spindled stroma, as originally described by Tsui et al. in the first description of biliary adenofibromata. At that time, the similarity to von Meyenberg complexes was observed. Furthermore, bile pigment in the tumor ducts implied a direct continuity with the biliary system. Parada et al. reported monosomy 22 in a case of biliary adenofibroma in a 49-year-old woman. However, this finding was not consistently found in a subsequent report [4].

Malignant transformation of biliary adenofibromata has been described in two previous cases. Akin described a 25-year-old male who demonstrated a recurrent biliary adenofibroma with pulmonary metastasis two years after initial resection. However, histologic findings of the malignant transformation were not described. Nguyen et al. described a biliary adenofibroma with multiple foci of epithelial atypia, including cytologic atypia and architectural atypia (cribriforming), representing high-grade dysplasia. They noted a microinvasive carcinoma (<1 mm) with increased atypia, including mildly enlarged epithelial cells with prominent nucleoli, a rare single cell in the stroma, and disrupted glands that merged with sclerotic stroma.

**Figure 4:**
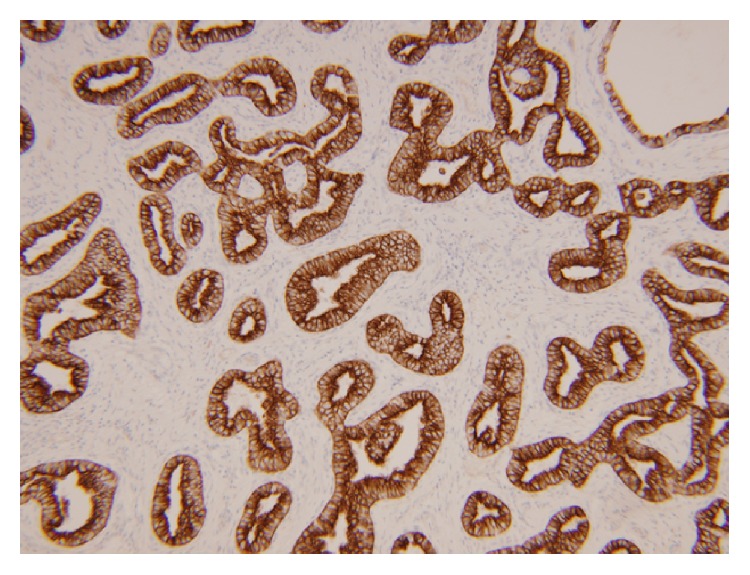
Conventional biliary adenofibroma, CK7, 10x.

**Figure 5:**
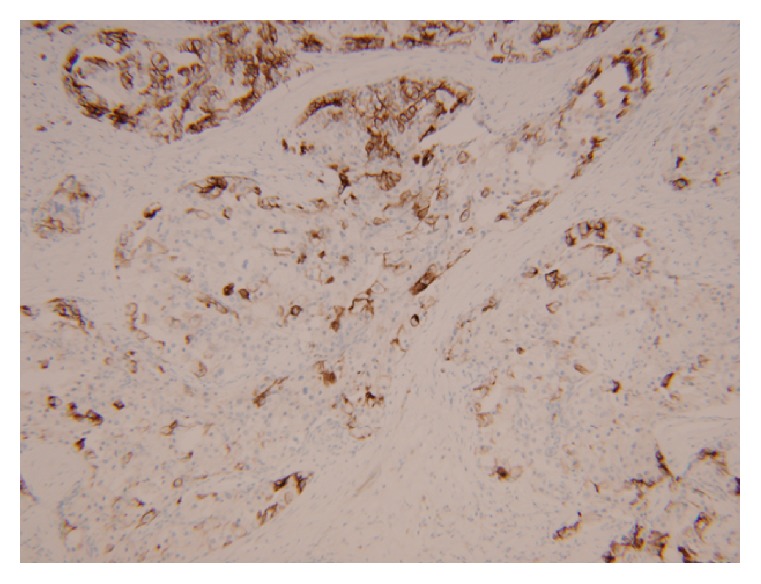
Invasive carcinoma, CK7, 10x.

Our malignant component shows distinct histology and demonstrates nearly solid architecture. Immunohistochemical findings showed variable reactivity between the benign and malignant components. The bland tubulocystic glands of the conventional biliary adenofibroma stained strongly and homogeneously positive for cytokeratins 7 and 19, while the carcinomatous areas showed weaker and more variable staining for these cytokeratins (Figures 4 and 5). P53 has been reported in varying rates of positivity ranging from negative to 75% of cells displaying strong nuclear staining; however, case reports of biliary adenofibroma with malignant transformation have not included a description of p53 staining pattern [2, 3, 5, 6].

Although the origin of biliary adenofibromata is uncertain, there is undeniable histologic and immunohistochemical similarity to von Meyenberg complexes. Tsui et al. noted the resemblance to Meyenberg's complex; however, they described an absence of typical von Meyenberg complexes in the background liver. Varnholt et al. reported a case of biliary adenofibroma and furthermore characterized the tumoral glandular epithelium staining positive for D10, p53 (50–75% of the cells), keratin AE.3/Cam 5.2, cytokeratin 7, cytokeratin 19, carcinoembryonic antigen, and epithelial membrane antigen. They further noted that the epithelial lining did not stain for IF6. Bile duct adenomas and peribiliary glands express foregut antigens, designated D10 and IF6, and the secretion of acid mucins. (Normal bile ductules and canals of Hering demonstrate positivity for IF6 but not D10, whereas larger bile ducts may demonstrate D10 positivity in 22% of cases, with an absence of IF6 [7].)

A similar pattern of D10 positivity/IF6 negativity was reported in one bile duct hamartoma. Thus, Varnholt et al. concluded that although the origin of biliary adenofibroma is unknown, the epithelial expression of D10 but not IF6 suggests an origin similar to bile duct hamartoma [4].

von Meyenberg complexes are largely considered benign and indolent; however, neoplastic transformation has been rarely reported. The most common malignancy arising in the setting of ductal plate malformations (including von Meyenberg complexes, biliary cystic lesions, and congenital hepatic fibrosis) is cholangiocarcinoma. Hepatocellular carcinomas, adenosquamous carcinomas, squamous cell carcinomas, and papillomas have also been reported [8]. The carcinoma seen in our case is difficult to classify precisely; however, it shows unequivocal features of malignancy. A summary of published tumor characteristics is included in Table 1.

This case of biliary adenofibroma with invasive carcinoma significantly expands the understanding of this rare tumor. While the presence of a von Meyenberg complex in liver adjacent to tumor does not prove the cellular origin of the tumor, the identification of a von Meyenberg complex in sections adjacent to tumor supports the observation that these tumors may share similar histogenesis as its immunomorphologic counterpart. Despite previous case reports describing a malignant transformation, biliary adenofibromata are currently generally regarded as benign entities. It is clear that they harbor potential for malignant transformation and therefore should be considered a premalignant tumor. P53 immunohistochemical stain has been performed in multiple cases in an effort to predict biological behavior. While the stromal component is consistently negative, the epithelial component demonstrates wide variability in staining and perhaps provides a mechanism to stratify risk of malignant transformation. As these tumors are poorly understood, patients require close clinical follow-up and observation.

## Figures and Tables

**Table 1:** Clinical and pathologic features of biliary adenofibromata reported in the literature.

	Year	Age/sex	Tumor size	Ki67%	Associated malignancy	Follow-up
Tsui et al. [1]	1993	74/F	7 cm	Not performed	No	

Parada et al. [9]	1997	49/F	7 cm	Not performed	No	

Akin and Coskun [2]	2002	25/M	20 cm	Not performed	Yes	Pulmonary metastasis 3 years after initial diagnosis

Haberal et al. [3]	2001	21/M	25 cm	Not performed	No	

Garduño-López [10]	2002	68/F	6 cm	Not performed	No	50-month follow-up

Varnholt et al. [4]	2003	47/F	16 cm	Ki67: low stromal component negative	No	3-year follow-up

Gurrera et al. [11]	2010	79/M	5.5 cm	Ki67 1% stromal and epithelial	No	7-year follow-up

Kai et al. [5]	2012	40/M	7 cm	Ki67 5–10%	Unclassified multicystic biliary tumor	

Nguyen et al. [6]	2012	53/F	6.5 cm	Not performed	Yes	12-month follow-up no recurrence

Godambe et al. (present case)	2013	71/F	5.7 cm	Ki67 50% epithelial component	Yes	
